# Repeated 28-Day Oral Toxicological Study and Gastroprotective Effects of *Nigella sativa* L. Oil (Shuhada) against Ethanol-Induced Gastric Mucosal Injury in Rats

**DOI:** 10.3390/nu15061532

**Published:** 2023-03-22

**Authors:** Sineenart Sanpinit, Palika Wetchakul, Piriya Chonsut, Ngamrayu Ngamdokmai, Aktsar Roskiana Ahmad, Sakan Warinhomhoun

**Affiliations:** 1 Division of Applied Thai Traditional Medicine, School of Medicine, Walailak University, Nakhon Si Thammarat 80160, Thailand; 2 Research Center in Tropical Pathobiology, Walailak University, Nakhon Si Thammarat 80160, Thailand; 3 Center of Excellence in Marijuana, Hemp, and Kratom, Walailak University, Nakhon Si Thammarat 80160, Thailand; 4 Department of Pharmacognosy and Phytochemistry, Faculty of Pharmacy, Universitas of Muslim Indonesia, Makassar 90241, South Sulawesi, Indonesia

**Keywords:** traditional Arabic and Islamic medicine, black cumin seed oil, thymoquinone, Quran, gastric ulcer, toxicity

## Abstract

*Nigella sativa* L. and black seeds are traditionally used for cooking and medicinal purposes in Arab and other countries. Although *N. sativa* seed extract has many known biological effects, the biological effects of cold-pressed *N. sativa* oil are poorly understood. Therefore, the objective of this study was to investigate the gastroprotective effects and subacute oral toxicity of black seed oil (BSO) in an animal model. The gastroprotective effects of oral BSO (50% and 100%; 1 mg/kg) were tested using acute experimental models of ethanol-induced gastric ulcers. Gross and histological gastric lesions, ulcerated gastric areas, ulcer index score, percentage of inhibition rate, gastric juice pH, and gastric wall mucus were all evaluated. The subacute toxicity of BSO and its thymoquinone (TQ) content were also examined. The results indicated that the administration of BSO exerted gastroprotective effects by increasing the gastric wall mucus and decreasing gastric juice acidity. In the subacute toxicity test, the animals behaved normally, and their weight and water and food intake did not show significant variations. High-performance liquid chromatography detected 7.3 mg/mL TQ in BSO. These findings suggest that BSO may be a safe therapeutic drug for preventing gastric ulcers.

## 1. Introduction

According to the World Health Organization (WHO), approximately 70% to 80% of the world’s population relies on traditional medicine for health care. Herbal treatments are the most widely used traditional medicine [1]. One example is *Nigella sativa* L., also known as black seed, a member of the Ranunculaceae family. It is an important plant in both Christian and Muslim traditions, owing to its religious significance [2], and Prophet Muhammad described the healing abilities of black seeds with the statement “Hold on to use this black seed, as it contains a treatment for every ailment save death” [3]. Black seed oil (BSO) has traditionally been used to treat a variety of ailments, diseases, and problems affecting the immune, cardiovascular, and digestive systems, and overall well-being. Various bioactive compounds, including thymoquinone (TQ), indazole alkaloids, flavonoids, phenolic compounds, saponins, and many others, are found in BSO, which are effective in treating patients with a variety of diseases [4]. Several previous studies evaluated the anti-gastric ulcer activity of *N. sativa* extract (NSE) and *N. sativa* oil (NSO) in mice or rat models induced by various concentrations of ethanol. In 2000, El-Dakhakhny reported that NSO had gastroprotective action against a single dose 50% ethanol-induced gastric ulcer in rats, through increasing the glutathione level and mucin content [5]. Kanter et al. (2005–2006) reported that NSO prevented single-dose 100% ethanol-induced gastric ulcer in rats through an increased gastric glutathione content (GSH) and the antioxidant enzymatic activities of gastric superoxide dismutase (SOD) and glutathione-S-transferase (GST). In addition, the NSO has also antihistaminic effects [6,7]. In 2010, El-Masry showed that NSO treated gastric mucosal lesions induced by various doses of ethanol through increasing antioxidant enzymes (SOD, GST, and glutathione peroxidase; GPx) [8].

Despite the general public’s notion that herbal treatments are safe, their toxicity remains a significant barrier to their use. To prevent the public from being exposed to harmful phytochemicals, the WHO recommends that herbal treatments undergo thorough scientific testing of both their efficacy and toxicity [9].

Gastric ulcers, one of the most prevalent disorders, are formed when there is an imbalance of protective and destructive factors in the gastric system. The gastric mucosa is frequently exposed to harmful substances, such as alcohol, caffeine, medications, pepsin, gastric acid, and bacteria [10]. One of these factors, high alcohol consumption, causes the most harm to the stomach mucosa, by destroying the gastric mucus, increasing gastric acidity, and increasing inflammatory cell infiltration into ulcers. Therefore, experimental models of ethanol-induced gastric ulcers are frequently used [11].

The four main categories of toxicity research are acute, subacute, chronic, and sub-chronic. Subacute toxicity, often known as repeat-dose toxicity, is associated with adverse effects following the administration of multiple daily doses or continuous exposure of a test sample over a period of approximately 28 days [12].

Although much work has been done on the gastroprotective activity of NSO and BSO, that research collected raw material of *N. sativa* seeds from various countries such as Egypt, Turkey, India, and Ethiopia [5,6,7,8]. Our literature review revealed a lack of raw material from Saudi Arabia. Therefore, the present study aimed to determine the effect of raw material BSO from Saudi Arabia, using a gastroprotective and subacute toxicity test in an animal model.

## 2. Materials and Methods

### 2.1. Preparation of BSO

The BSO was from Shuhada Thailand Co., Ltd. (Bangkok, Thailand), who import the raw material from Saudi Arabia.

### 2.2. Experimental Animals

Healthy male and female Wistar albino rats (*Rattus norvegicus*), aged 6 weeks (150–200 g), were obtained from Nomura Siam International Co., Ltd. (Bangkok, Thailand). The rats were allowed to acclimatize for 7 days with ad libitum access to standard pellets and clean drinking water. All animals, grouped by sex, were kept in polycarbonate cages and maintained at a temperature of 23 °C ± 2 °C and relative humidity of 45% to 55% under a 12 h natural light/dark cycle within the Laboratory Animal Unit of the Research Institute for Health Sciences of Walailak University.

### 2.3. Ethanol-induced Gastric Ulceration

Male rats were randomly allocated into four groups (*n* = 6):

1 mL/kg body weight (BW) distilled water-treated + ethanol-treated group (control group),
20 mg/kg omeprazole + ethanol-treated group (positive group),
1 mL/kg BW of 100% BSO + ethanol-treated group (H-BSO group),
1 mL/kg BW of 50% BSO + ethanol-treated group (L-BSO group)

Omeprazole and BSO were administered orally once daily for 7 days, before ulcer induction. On day 7, after treatment with omeprazole and BSO for approximately 4 h, all rats were subjected to oral gavage with 80% alcohol (1 mL/kg). One hour later, the rats were sacrificed using an overdose of sodium thiopental. Blood samples were collected for white blood cell (WBC) and differential WBC counts. In parallel, animal stomachs were rapidly removed and opened along the greater curvature, and the gastric juice pH was determined using a pH indicator strip.

### 2.4. Gross Evaluations of Gastric Tissue

The gastric tissue was then opened along the greater curvature and rinsed with normal saline, to remove gastric contents. The gastric tissues were flattened on plasticine and fixed with a pin for photography using a digital camera. The ulcerated areas were evaluated using Image J 1.48v, a Java-based image processing program (National Institutes of Health, Bethesda, MD, USA), and the results were expressed in terms of the percentage of the inhibition range of the induced ulceration.

Percentage of the inhibition range = (ulcer area of control − ulcer area of treated)/(ulcer area of control) × 100(1)

The ulcer index (UI) was measured using the following lesion scores: 0, normal colored stomach; 0.5, red coloration; 1, spot ulcer; 1.5, hemorrhagic streak; 2, deep ulcers; 3, perforation. To determine the UI, the total number of lesions was divided by the total score for each lesion. Next, the ulcerated portions were carefully cut, for the measurement of mucus content and for histopathological analysis.

### 2.5. Measurement of Gastric Mucus Content

The glandular portion of gastric tissues was weighed and transferred to a small bottle with a lid containing 10 mL 0.1% Alcian blue solution prepared in 0.16 M sucrose and 0.05 M sodium acetate (pH 5.0) for 2 h at room temperature and then washed twice with 0.25 M sucrose solution for 15 and 45 min. MgCl_2_ (0.5 M, 5 mL) was added to each bottle, to extract the dye complexed with the gastric mucus for approximately 2 h. During this period, the soaked stomach was shaken for 1 min every 30 min. A 4 mL aliquot of the blue extract was then shaken with an equal volume of diethyl ether, until the formation of an emulsion, and was centrifuged at 4000 rpm for 10 min. The absorbance of the supernatant (blue solution) was measured at a wavelength of 605 nm. The concentration of Alcian blue (weight (µg) of Alcian blue per gram of tissue) was calculated using a standard calibration curve.

### 2.6. Histopathology of Gastric Tissue

Small pieces of gastric tissue were fixed in 4% paraformaldehyde for 24 h. The washed tissues were embedded in paraffin blocks and cut into 5 μm sections. The sections were stained with hematoxylin and eosin (H&E). The tissue sections were observed under a microscope, and images were analyzed using AxioVision digital image processing software (Zeiss, Jena, Germany), to characterize histopathological changes, such as congestion, hemorrhage, edema, and erosion.

### 2.7. Assessment of 28-day Repeated-Dose Oral Toxicity

The 28-day repeated-dose toxicity method was performed according to the protocol suggested in OECD Guideline 407 (2008). Wistar rats of both sexes were randomly assigned to three experimental groups, consisting of 12 rats per group (6 males and 6 females): control group, which was given 1 mL/kg BW of distilled water; H-BSO group, which was given 1 mL/kg BW of H-BSO orally; L-BSO group, which was given 1 mL/kg BW of L-BSO orally. Dosing was performed once daily in the morning for 28 days. Behavioral patterns and physical appearance, in addition to other adverse effects, such as lethargy, diarrhea, tremors, salivation, etc., were monitored in a timely manner. Individual animal weight and feed consumption were measured daily during the dosing period. Food and water intake were measured as grams per day per group and milliliters per day per group, respectively. During the experiment, the body weight of the rats was determined using a digital weighing balance (daily). On day 29, the animals were fasted for 16 h with free access to water and then anesthetized and euthanized for collection of blood samples and vital organs.

### 2.8. Assessment of Biochemical Parameters

The blood samples were analyzed for biochemical parameters, including blood urea nitrogen (BUN), creatinine, cholesterol, triglyceride, high- and low-density lipoproteins (HDL and LDL, respectively), total protein, albumin, total bilirubin, aspartate aminotransferase (AST), alanine aminotransferase (ALT), and alkaline phosphatase.

### 2.9. Assessment of Hematological Parameters

Blood samples were analyzed for hematological parameters, including red blood cells, hemoglobin, hematocrit, mean corpuscular volume, mean corpuscular hemoglobin, mean corpuscular hemoglobin concentration, red blood cell distribution width, platelet count, WBC, neutrophils, lymphocytes, eosinophils, basophils, and monocytes.

### 2.10. Relative Weight of Organs

The vital organs, including the heart, liver, kidneys, lungs, spleen, and testis/ovaries, were carefully removed from the dissected rats, washed with distilled water, and weighed. The organs were examined for abnormalities and changes in size, color, consistency, and texture. Relative organ weights were determined as follows:

Relative organ weight = (weight of organ)/(body weight of the rat on the sacrifice day) × 100(2)

### 2.11. H&E Staining of Vital Organs

All vital organs were preserved in 10% formalin, dehydrated, embedded, sectioned, mounted, and counterstained with H&E; then, histopathologic examinations were performed.

### 2.12. Quantification of TQ Using High-Performance Liquid Chromatography

The high-performance liquid chromatograph (HPLC; UltiMate™ 3000; Thermo Fisher Scientific, Waltham, MA, USA) used in this study was equipped with a U3000 DAD detector and automatic injector. The reversed-phase column used was a VertiSep™ USP C18 4.6 × 250 nm (5 µm) (Vertical Chromatography Co., Ltd., Nonthaburi, Thailand) controlled at 37 °C, with gradient elution at a flow rate of 1.0 mL/min. The injection volume was 20 µL. The gradient was initiated with 75% acetonitrile (Solvent A) and 1% *v*/*v* acetic acid (Solvent B) for 12 min, then automatically changed to 70% (Solvent A), 10% (Solvent B), and 20% methanol (Solvent C) for 8 min, and automatically decreased to the initial mobile phase ratio for 10 min. Detection was performed at a wavelength of 254 nm.

### 2.13. Statistical Analysis

Data were analyzed using Statistical Package for Social Science version 23 (IBM Corp., New York, NY, USA). Analysis of variance was conducted to compare data variations between and within the groups. Post hoc analysis using Duncan’s test was performed. Results were considered statistically significant at *p* < 0.05.

## 3. Results

### 3.1. Effects of BSO on Gross Gastric Mucosal Injury

**Figure 1:**
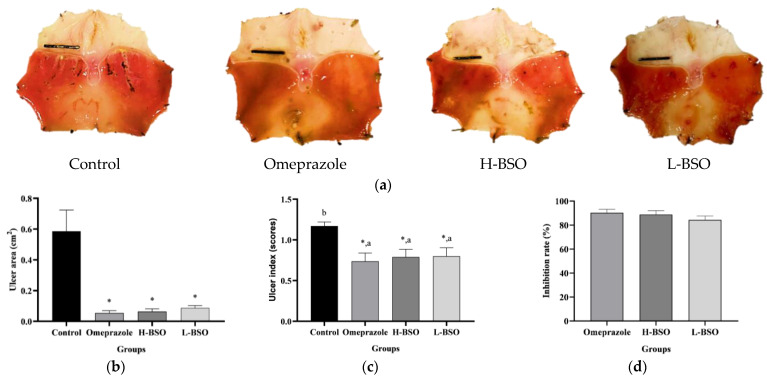
Gross gastric lesions (**a**), ulcer gastric areas (**b**), ulcer index score (**c**), and percentage of inhibition rate (**d**) in rats with ethanol-induced gastric ulcers pretreated with distilled water, omeprazole, 100% black seed oil (H-BSO), and 50% black seed oil (L-HSO). * Significantly different from the control at *p* < 0.05. Different letters (^a–b^) indicate significant differences (*p* < 0.05) between groups determined by Duncan’s multiple-range test. Scale bar = 1 cm.

Oral administration of 80% ethanol induced gross lesions in the gastric lumen of rats, with a markedly high ulceration index. The rats pretreated with distilled water had more damaged lesions than the others, with severe hemorrhagic lesions, erosion, and hyperemia (Figure 1a). In contrast, the rats that received H-BSO and L-BSO showed significantly reduced gastric ulcer areas, similarly to the pretreatment with omeprazole (Figure 1b). The ulcer indices of the rats pretreated with omeprazole, H-BSO, and L-BSO were significantly lower than those of the control group (Figure 1c). Moreover, the ulcer inhibition rates were 88.33% and 83.45% after pretreatment with H-BSO and L-BSO, respectively. Omeprazole, the standard drug, exhibited a higher ulcer inhibition percentage than BSO (88.84%); however, the difference was not significant (Figure 1c).

### 3.2. Effects of BSO on Gastric Juice pH and Gastric Wall Mucus

Omeprazole and L-BSO pretreatment in ethanol-induced ulcerated rats significantly increased the pH value (3.67 ± 0.21 and 3.50 ± 0.22) compared with ethanol-induced ulcerated rats without pretreatment (2.33 ± 0.21) (Table 1). The gastric wall mucus content was significantly increased to 21.33 ± 2.23, 22.64 ± 1.15, and 21.64 ± 0.66 μg Alcian blue/g of tissue in the ethanol-induced ulcerated rats that were given omeprazole, H-BSO, and L-BSO, respectively, compared with that of the control group (16.95 ± 0.40 μg Alcian blue/g of tissue) (Table 1).

### 3.3. Gastric Ulcer Model Pretreated with BSO

The 80% ethanol-exposed gastric ulcer in the control group showed a significant increase in WBC, neutrophil, and lymphocyte counts. Pretreatment with H-BSO, L-BSO, and omeprazole 20 mg/kg for 7 days resulted in a significant decrease in WBC, neutrophils, and lymphocytes compared with those of the control group (Table 2). Eosinophils and monocytes were not detected in the control group. In contrast, pretreatment with H-BSO, L-BSO, or omeprazole did not cause eosinophil or monocyte accumulation after ethanol-induced gastric ulcers.

### 3.4. Effects of BSO on Gastric Ulcer Evaluated by Histopathology

**Figure 2:**
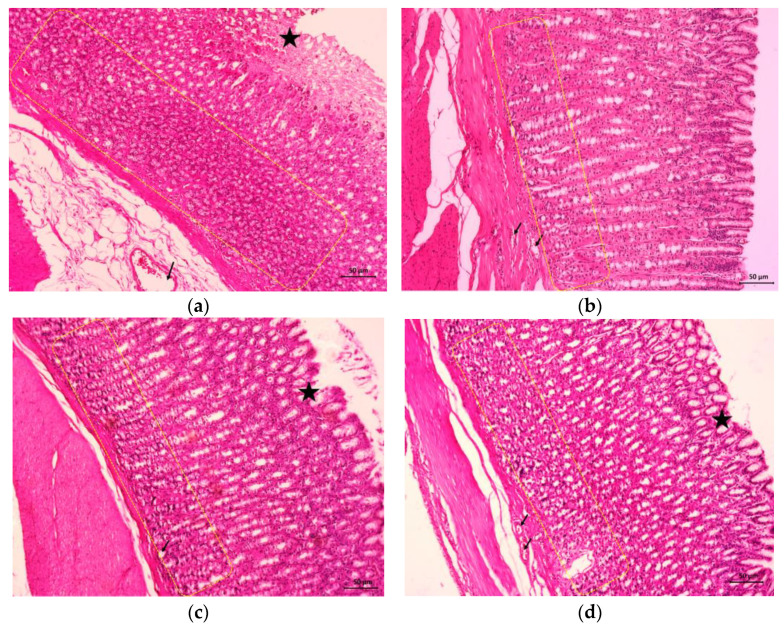
Histological study of ethanol-induced gastric ulcer in rats pretreated with 1 mL/kg of distilled water (control group) (**a**), 20 mg/kg of omeprazole (positive group) (**b**), 1 mL/kg of H-BSO (**c**), and 1 mL/kg of L-BSO. (**d**) Black star, disruption to the surface epithelium; black arrow, congested or dilated blood vessel; yellow dotted border, inflammatory cell infiltrations in the mucosal layer (hematoxylin and eosin, magnification 10×).

Figure 2a shows severe disruption of the surface epithelium (black star), very dense inflammatory cell infiltration of the mucosal layer (yellow dotted border), and extensive edema and vasodilation (black arrow) of the submucosa layer with acute oral administration of 80% ethanol-induced gastric ulcers in the control group. Meanwhile, pretreatment of the gastric tissues with 20 mg/kg omeprazole demonstrated no disruption of the surface epithelium, no edema of the submucosa layer, and only a light infiltration of inflammatory cells in the mucosal layer (Figure 2b). Similarly, pretreatment with H-BSO and L-BSO resulted in minimal disruption to the surface epithelium and few infiltrations of inflammatory cells in the mucosal layer when compared with the control group (Figure 2c,d).

### 3.5. Effects of BSO on Subacute Oral Toxicity

#### 3.5.1. Clinical Signs and Mortality

The effects of H-BSO and L-BSO on clinical signs and mortality are presented in Table 3. All clinical signs, including pain, stress, abnormal behavior, physical changes, and mortality, were absent in all groups of rats of both sexes within 28 days of the study and observation.

#### 3.5.2. Body Weight, and Food and Water Consumption

**Figure 3:**
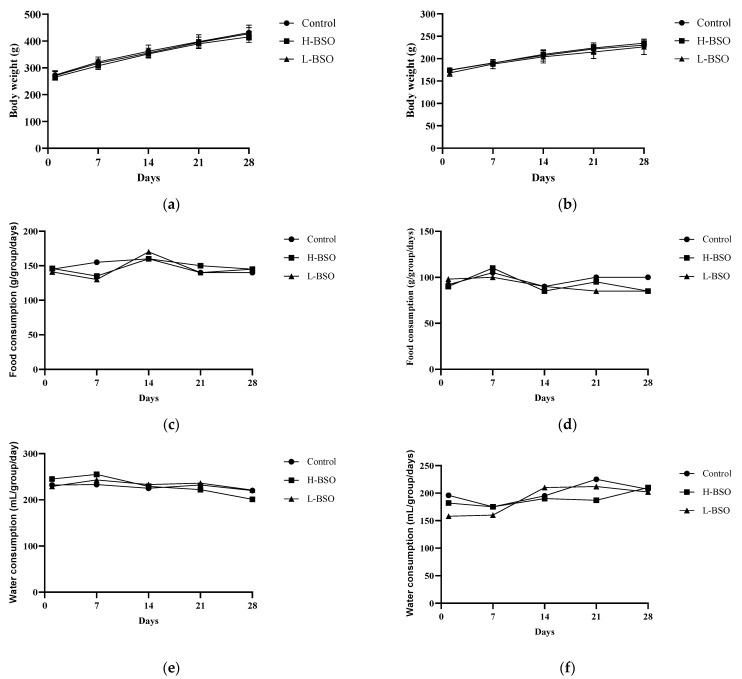
Body weight, food consumption, and water consumption of rats ((**a**,**c**,**e**): male; (**b**,**d**,**f**): female) after administration of H-BSO and L-BSO for 28 days. Values are expressed as ± standard error of the mean; one-way analysis of variance followed by Tukey test (*n* = 6 animals/group). Differences between groups were considered to be significant at *p* < 0.05.

The body weights of the male and female rats obtained at the beginning of the experiment (day 0) and at days 7, 14, 21, and 28 after H-BSO and L-BSO administration did not show any significant (*p* > 0.05) changes compared with those of the control group (Figure 3a,b). No change was observed in the food and water consumption patterns of male and female rats treated with H-BSO and L-BSO compared with those of the control group (Figure 3c–f).

#### 3.5.3. Biochemical Analysis

All biochemical parameters, including indicators of hepatocellular effects (such as ALT, AST, bilirubin, and albumin), lipid profile (such as cholesterol, triglyceride, HDL, and LDL), and biomarkers of nephron functional injury (BUN and creatinine), were not significantly different between the control group and male rats that were given H-BSO and L-BSO. However, in female rats, significant differences were observed for some biochemical parameters, including decreased cholesterol and increased albumin, after administration of H-BSO and L-BSO for 28 days, compared with the control group (Table 4).

#### 3.5.4. Hematological Analysis

Data of the hematological parameters in the control, H-BSO, and L-BSO groups with subacute oral toxicity are presented in Table 5. There were no significant differences in hematological parameters between the male and female rats.

#### 3.5.5. Relative Vital Organ Weight

There were no significant differences (*p* > 0.05) observed in the organ weights of the heart, liver, kidney, lungs, spleen, or sex organs (testis/ovary and uterine tube) relative to body weight of the rats among the different groups compared with the control group (Table 6).

#### 3.5.6. Histopathology of Vital Organs

**Figure 4:**
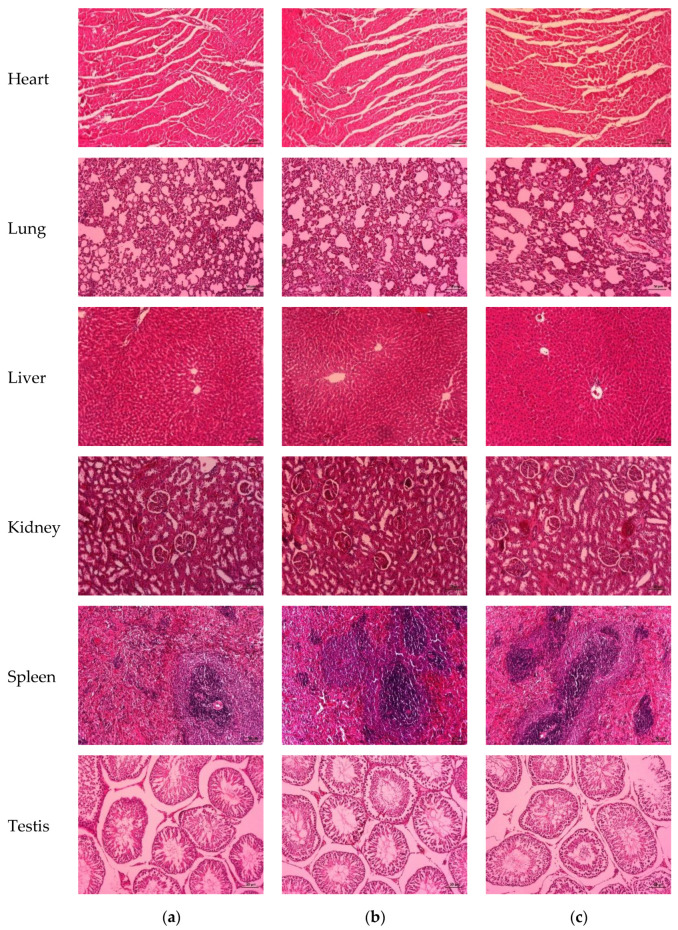
Histopathological analysis of the vital organs of male rats treated with distilled water (**a**), H-BSO (**b**), and L-BSO (**c**) for 28 days showed normal morphology (hematoxylin and eosin, magnification 10×). Scale bar = 50 μm.

**Figure 5:**
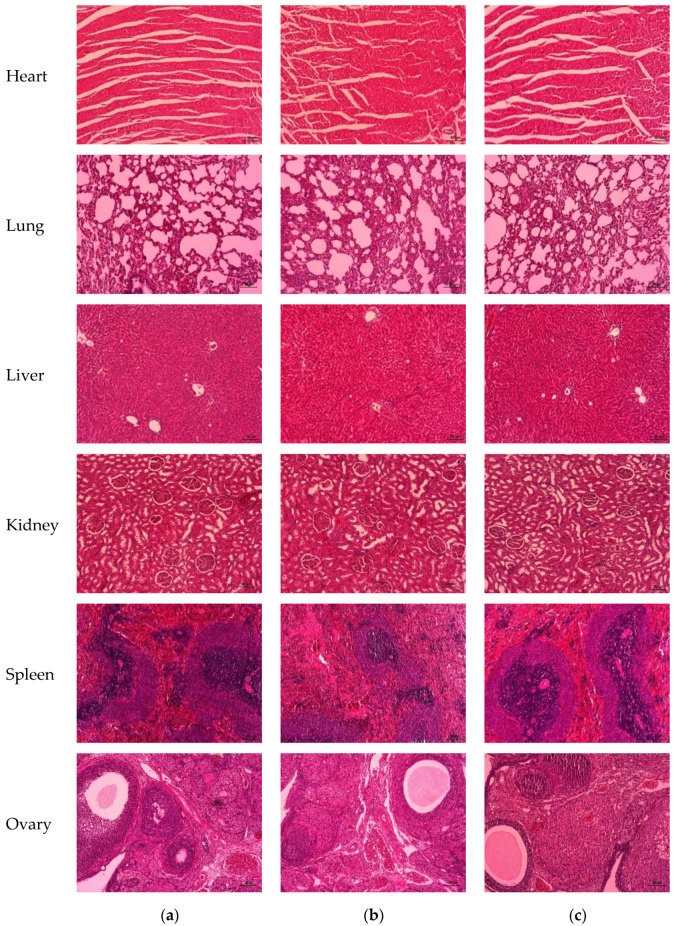
Histopathological analysis of the vital organs of female rats treated with distilled water (**a**), H-BSO (**b**), and L-BSO (**c**) for 28 days showed normal morphology (hematoxylin and eosin, magnification 10×). Scale bar  =  50 μm.

The histopathological analyses of the vital organs, including the heart, lung, liver, kidney, spleen, and reproductive organs, of the rats (male and female rats) after administration of H-BSO and L-BSO for 28 days showed normal structures, and there was no alteration in the cell structure or any negative effect on the organs compared with those of the control group (Figure 4 and Figure 5).

#### 3.5.7. TQ Contents in BSO

**Figure 6:**
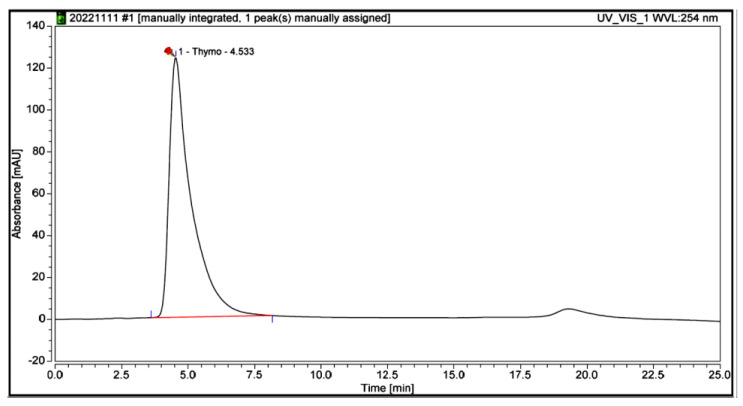
High-performance liquid chromatogram of standard thymoquinone solution.

**Figure 7:**
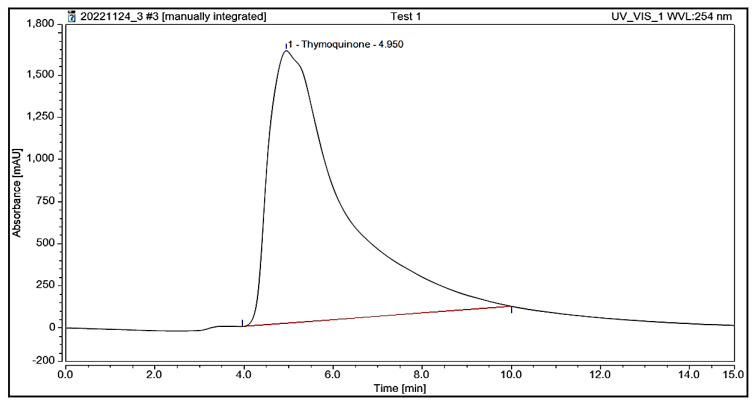
High-performance liquid chromatogram of thymoquinone in black seed oil.

The TQ content in BSO, as determined through HPLC analysis, was 7.3 mg/mL. Figure 6 and Figure 7 show the HPLC of the standard thymoquinone solution and thymoquinone in BSO, respectively.

## 4. Discussion

*N. sativa* L. or *hubatul-sudda* (Arabic name) is widely used in traditional Arabic and Islamic, Ayurvedic, and Iranian traditional medicine, as well as in traditional folk medicine in Thailand. The lower southern part of Thailand has a large Muslim population, who believe in the use of various herbs for medical purposes, as described in the Quran. The hadith literature, compiled during the eighth and ninth centuries and attributed to the Islamic Prophet Muhammad, also details the medicinal uses of *N. sativa*. In traditional Arabic and Islamic medicine practice, a variety of gastrointestinal ailments are treated using both seeds (eaten with honey) and fatty oil. This study analyzed the properties and oral safety of *N. sativa* L. and BSO.

Gastric ulcers result from a disturbance in the balance between protective factors, including gastric mucin, bicarbonate, or endogenous antioxidants, and destructive factors, including infections, increased acid production, and alcohol consumption, in the gastric system [13]. The pathophysiology of stomach ulceration and the assessment of the gastroprotective effects of various medications and natural items have been studied using the ethanol-stimulated gastric ulcer model [14]. In this study, we used this model to evaluate the gastroprotective effects of BSO. Our findings showed that pretreatment with H-BSO and L-BSO for 7 days reduced the gastric ulcer area, UI score, and gastric acidity, and simultaneously restored the levels of gastric wall mucus in ethanol-induced gastric ulcers. A previous study showed that pretreatment with 0.88 g/kg BW of BSO (raw material from Egypt) for 2 weeks produced an ulceration inhibition rate of about 53% [5], but our results showed pretreatment with 1 mL/kg of 50% and 100% BSO (raw material from Saudi Arabia) for only 1 week showed a high ulceration inhibition rate of about 80%. In addition, the raw material of N. sativa seed from Turkey showed an inhibition rate of gastric ulceration of about 60% [6,7]. This finding demonstrated that the raw material of *N. sativa* seed from different planting areas results in different gastroprotective properties. Therefore, it can be assumed that N. sativa seed from Saudi Arabia have a high gastroprotective effect in the ethanol-induced gastric ulcer rat model. Several studies have shown that stimulated production of gastric wall mucus and reduced gastric acidity prevent ethanol-induced gastric ulcers [14,15,16,17]. It was hypothesized that BSO can effectively reduce the gastric ulcer area by stimulating the production of gastric wall mucus and suppressing gastric acidity, compared with the control pretreatment (distilled water).

In addition, an imbalance between protective and destructive factors or increased gastric acidity, reduction of stomach blood flow, induction of oxidative stress, and increased attraction of inflammatory cells are the mechanisms by which ethanol causes gastric ulcers [18]. The results of the current study indicate that the control group, which received distilled water and ethanol, had the highest number of total WBCs in the blood sample. In contrast, WBCs, including neutrophils, lymphocytes, eosinophils, and monocytes, decreased in the rats pretreated with omeprazole, H-BSO, and L-BSO. These findings agree with those of the histopathology study, which showed that the control group had very dense inflammatory cell infiltration of the mucosal layer. Conversely, rats pretreated with omeprazole and BSO showed light infiltration of inflammatory cells in the ulcer gastric tissue. Several studies have demonstrated the anti-inflammatory effects of BSO. Attia et al. (2016) reported the anti-inflammatory effect of BSO 400 mg/kg in a carrageenan-induced rat paw edema model [19]. Dwita et al. (2019) evaluated the anti-inflammatory effect of BSO balm stick on carrageenan-induced paw edema and granuloma pouch in rats and reported a 43.55% reduction in leukocyte count compared with that of the control group [20]. The major bioactive compound in BSO behind its anti-inflammatory activity is TQ. In the present study, the TQ content in BSO was 7.3 mg/mL, which is higher than those reported by previous studies [21,22]. TQ reduced the levels of pro-inflammatory cytokines in lipopolysaccharide-stimulated murine macrophage-like RAW264.7 cells in earlier investigations [23,24]. Our results suggest that the TQ in BSO may have anti-inflammatory properties in ethanol-induced gastric ulcers.

The toxicological profiles of BSO have also been studied [25]. Our study used different BSO planting sites and oil extraction methods than previous studies. Therefore, subacute oral toxicity studies should be conducted, to confirm the safety of BSO, as well as the toxic response of BSO to targeted organs of the body. Our findings demonstrated that both sexes of rats administered H-BSO and L-BSO during a 28-day experiment exhibited no signs of toxicity, and no mortality was recorded. Comparison of the BSO treatment group with the control group showed no significant variation in the mean body weight, food water consumption, and relative vital organ weights, indicating that BSO may not have toxic effects. In addition, no significant difference was observed in the hematological analysis and renal function parameters of both sexes of rats compared with the control group. Biochemical analysis showed no significant difference in rats of both sexes compared with the control group. However, cholesterol levels were significantly decreased in the female rats treated with BSO compared with those in the control group. Moreover, there was a slight nonsignificant decrease in triglyceride and LDL levels, suggesting that BSO may reduce the production of LDL and triglycerides in the liver. The results of the hematological and biochemical analyses are supported by a previous histopathological study [12]. The results of the histopathological analysis of the heart, liver, kidney, lungs, spleen, and sex organs indicated no modifications in the tissue architecture of the organs after repeated 28-day oral administration of BSO, which supports our belief that BSO is safe and nontoxic. However, future studies must be performed to assess the safety of long-term BSO oral administration, such as chronic toxicity tests, for application to functional food products.

## 5. Conclusions

In light of these findings, we conclude that BSO had a significant protective effect against gastric ulcers in rats, which have may been due to its ability to stimulate the production of gastric wall mucus and reduce gastric acidity in a rat model of ethanol-induced gastric ulcers. These findings support the traditional use of BSO in the Quran. The results of the present in vivo subacute toxicity study suggest that oral administration of BSO does not cause adverse effects in rats of either sex. Therefore, the findings of the current study on the gastroprotective effect and toxicity suggest that BSO may be used as a reliable and safe herbal medicine or functional food. Exploration of the underlying mechanisms responsible for the gastroprotective activity of BSO will be the focus of our future research.

## Figures and Tables

**Table 1:** Effect of orally administered distilled water, omeprazole, H-BSO, and L-BSO on pH value and gastric wall mucus production in the ethanol-induced gastric ulcer model.

Groups	pH Value	Mucin Content (μg Alcian Blue/g of Tissue)
Control	2.33 ± 0.21 ^a^	16.95 ± 0.04 ^b^
Omeprazole	3.67 ± 0.21 *^,b^	21.33 ± 2.23 *^,a^
H-BSO	2.50 ± 0.22 *^,a^	22.64 ± 1.15 *^,a^
L-BSO	3.50 ± 0.22 *^,b^	21.64 ± 0.66 *^,a^

Data are presented as mean ± standard error of the mean (*n* = 6) per group and were analyzed using one-way analysis of variance and post hoc Tukey test. * Significant differences compared with the control group (*p* < 0.05). ^a,b^ Significant differences (*p* < 0.05) between groups determined by Duncan’s multiple-range test.

**Table 2:** White blood cell count (WBC) and differential WBC in blood of rats pretreated with distilled water, omeprazole, H-BSO, and L-BSO in the ethanol-induced gastric ulcer model.

Groups/Parameters	Control	Omeprazole	H-BSO	L-BSO
WBC (cells/mm^3^)	6825.00 ± 832.04 ^b^	5875.00 ± 535.99 ^ab^	4250.00 ± 259.81 ^a^	5275.00 ± 464.35 ^ab^
Neutrophils (%)	17.00 ± 2.27 ^b^	11.00 ± 1.73 ^a^	11.25 ± 0.48 ^a^	15.25 ± 0.85 ^ab^
Lymphocyte (%)	86.50 ± 0.87 ^b^	85.75 ± 0.25 ^ab^	86.50 ± 0.87 ^b^	83.00 ± 0.41 ^a^
Eosinophils (%)	0.33 ± 0.21 ^a^	0.17 ± 0.10 ^a^	0.17 ± 0.10 ^a^	0.00 ± 0.00 ^a^
Monocytes (%)	0.17 ± 0.00 ^a^	0.00 ± 0.00 ^a^	0.00 ± 0.00 ^a^	0.00 ± 0.00 ^a^
Basophils (%)	0.00 ± 0.00 ^a^	0.00 ± 0.00 ^a^	0.00 ± 0.00 ^a^	0.00 ± 0.00 ^a^

Values are presented as mean ± standard error of the mean. Means in the same row as the control group with different superscript letters are significantly different. *p* < 0.05, Tukey HSD.

**Table 3:** Clinical signs and mortality of rats treated with BSO for 28 days.

Groups	Total Animals	Observed Clinical Signs	Duration of Clinical Signs (Days)	Mortality
Male				
Control	6	Nil	28	0/6
H-BSO	6	Nil	28	0/6
L-BSO	6	Nil	28	0/6
Female				
Control	6	Nil	28	0/6
H-BSO	6	Nil	28	0/6
L-BSO	6	Nil	28	0/6

**Table 4:** Biochemical parameters of male and female rats treated with BSO for 28 days.

Groups/Parameters	Control	H-BSO	L-BSO
Male			
Blood urea nitrogen (mg/dL)	18.67 ± 0.33 ^a^	17.83 ± 0.65 ^a^	18.83 ± 0.82 ^a^
Creatinine (mg/dL)	0.39 ± 0.02 ^ab^	0.43 ± 0.03 ^b^	0.30 ± 0.01 ^a^
Cholesterol (mg/dL)	61.67 ± 2.26 ^a^	60.17 ± 1.17 ^a^	65.83 ± 2.95 ^a^
Triglyceride (mg/dL)	266.83 ± 24.48 ^a^	178.83 ± 28.72 ^a^	214.00 ± 25.33 ^a^
High-density lipoprotein (mg/dL)	53.83 ± 17.87 ^a^	55.17 ± 19.62 ^a^	66.33 ± 26.48 ^a^
Low-density lipoprotein (mg/dL)	45.67 ± 15.19 ^a^	43.00 ± 16.24 ^a^	45.33 ± 28.49 ^a^
Total protein (g/dL)	7.17 ± 0.02 ^a^	7.33 ± 0.17 ^a^	7.23 ± 0.56 ^a^
Albumin (g/dL)	3.12 ± 0.05 ^a^	3.08 ± 0.05 ^a^	3.17 ± 0.04 ^a^
Total bilirubin (mg/dL)	0.97 ± 0.10 ^a^	1.15 ± 0.22 ^a^	1.67 ± 0.35 ^a^
Aspartate aminotransferase (U/L)	183.00 ± 5.61 ^a^	186.17 ± 12.84 ^a^	195.33 ± 10.50 ^a^
Alanine aminotransferase (U/L)	36.67 ± 1.38 ^a^	36.67 ± 1.52 ^a^	34.50 ± 1.23 ^a^
Alkaline phosphatase (U/L)	90.83 ± 7.91 ^a^	94.17 ± 9.60 ^a^	90.56 ± 4.12 ^a^
Female			
Blood urea nitrogen (mg/dL)	18.17 ± 0.65 ^a^	18.00 ± 0.52 ^a^	18.67 ± 0.21 ^a^
Creatinine (mg/dL)	0.40 ± 0.04 ^a^	0.37 ± 0.03 ^a^	0.35 ± 0.02 ^a^
Cholesterol (mg/dL)	62.50 ± 1.06 ^b^	57.83 ± 1.25 ^a^	58.00 ± 1.26 ^a^
Triglyceride (mg/dL)	167.17 ± 29.19 ^a^	91.83 ± 8.31 ^a^	123.83 ± 18.63 ^a^
High-density lipoprotein (mg/dL)	41.33 ± 2.17 ^a^	42.67 ± 3.35 ^a^	41.00 ± 2.29 ^a^
Low-density lipoprotein (mg/dL)	13.33 ± 4.40 ^a^	9.67 ± 2.35 ^a^	10.17 ± 4.20 ^a^
Total protein (g/dL)	7.23 ± 0.03 ^a^	7.23 ± 0.02 ^a^	7.32 ± 0.01 ^a^
Albumin (g/dL)	2.98 ± 0.31 ^a^	3.43 ± 0.07 ^ab^	3.92 ± 0.06 ^b^
Total bilirubin (mg/dL)	0.98 ± 0.09 ^a^	0.83 ± 0.12 ^a^	1.03 ± 0.70 ^a^
Aspartate aminotransferase (U/L)	176.33 ± 6.28 ^a^	162.00 ± 10.15 ^a^	186.50 ± 11.03 ^a^
Alanine aminotransferase (U/L)	37.00 ± 1.37 ^a^	37.50 ± 1.54 ^a^	36.50 ± 1.52 ^a^
Alkaline phosphatase (U/L)	82.17 ± 7.78 ^a^	77.16 ± 6.88 ^a^	74.67 ± 6.30 ^a^

Values are presented as mean ± standard error of the mean. Means in the same row as the control group with different superscript letters are significantly different. *p* < 0.05, Tukey HSD.

**Table 5:** Hematological parameters of male and female rats treated with BSO for 28 days.

Groups/Parameters	Control	H-BSO	L-BSO
Male			
Red blood cells (×10^6^/µL)	7.42 ± 0.56 ^a^	7.35 ± 0.14 ^a^	7.34 ± 0.15 ^a^
Hemoglobin (g/dL)	15.62 ± 0.38 ^a^	15.38 ± 0.21 ^a^	15.43 ± 0.21 ^a^
Hematocrit (%)	44.67 ± 1.56 ^a^	44.60 ± 0.97 ^a^	44.60 ± 0.91 ^a^
Mean corpuscular volume (fL)	60.28 ± 0.58 ^a^	60.70 ± 0.86 ^a^	60.85 ± 0.51 ^a^
Mean corpuscular hemoglobin (pg)	21.03 ± 0.30 ^a^	22.55 ± 1.67 ^a^	20.98 ± 0.15 ^a^
Mean corpuscular hemoglobin concentration (g/dL)	34.97 ± 0.40 ^a^	34.50 ± 0.53 ^a^	34.57 ± 0.32 ^a^
Red blood cell distribution width (%)	15.58 ± 0.28 ^a^	15.11 ± 0.25 ^a^	15.42 ± 0.26 ^a^
Platelets (cells/mm^3^)	468,666.67 ± 9639.04 ^a^	503,333.33 ± 18,012.34 ^a^	493,000.00 ± 26,609.52 ^a^
White blood cells (cells/mm^3^)	4583.33 ± 791.38 ^a^	3650.00 ± 784.75 ^a^	2916.67 ± 628.98 ^a^
Neutrophils (%)	15.33 ± 1.98 ^a^	13.17 ± 0.95 ^a^	14.00 ± 0.93 ^a^
Lymphocyte (%)	84.66 ± 1.98 ^a^	86.67 ± 0.84 ^a^	86.00 ± 0.93 ^a^
Eosinophils (%)	0.00 ± 0.00 ^a^	0.00 ± 0.00 ^a^	0.00 ± 0.00 ^a^
Basophils (%)	0.00 ± 0.00 ^a^	0.00 ± 0.00 ^a^	0.00 ± 0.00 ^a^
Monocytes (%)	0.00 ± 0.00 ^a^	0.00 ± 0.00 ^a^	0.00 ± 0.00 ^a^
Red blood cells (×10^6^/µL)	6.95 ± 0.23 ^a^	6.55 ± 0.17 ^a^	7.70 ± 0.11 ^a^
Female			
Hemoglobin (g/dL)	15.18 ± 0.30 ^a^	14.15 ± 0.36 ^a^	15.02 ± 0.15 ^a^
Hematocrit (%)	42.85 ± 1.32 ^a^	40.25 ± 1.09 ^a^	43.62 ± 0.74 ^a^
Mean corpuscular volume (fL)	61.80 ± 0.60 ^a^	61.52 ± 0.71 ^a^	61.87 ± 0.84 ^a^
Mean corpuscular hemoglobin (pg)	21.85 ± 0.47 ^a^	21.53 ± 0.22 ^a^	21.27 ± 0.24 ^a^
Mean corpuscular hemoglobin concentration (g/dL)	35.47 ± 0.68 ^a^	35.11 ± 0.11 ^a^	34.30 ± 0.49 ^a^
Red blood cell distribution width (%)	14.47 ± 0.11 ^a^	14.90 ± 0.20 ^a^	14.62 ± 0.24 ^a^
Platelets (cells/mm^3^)	469,166.67 ± 18,120.73 ^a^	482,500.00 ± 10,101.98 ^a^	472,000.00 ± 16,194.65 ^a^
White blood cells (cells/mm^3^)	2933.33 ± 170.62 ^a^	2383.33 ± 246.87 ^a^	3083.33 ± 745.39 ^a^
Neutrophils (%)	14.33 ± 1.78 ^a^	20.00 ± 1.73 ^a^	16.00 ± 2.11 ^a^
Lymphocyte (%)	85.50 ± 1.64 ^a^	80.00 ± 1.73 ^a^	84.00 ± 2.11 ^a^
Eosinophils (%)	0.00 ± 0.00 ^a^	0.00 ± 0.00 ^a^	0.00 ± 0.00 ^a^
Basophils (%)	0.00 ± 0.00 ^a^	0.00 ± 0.00 ^a^	0.00 ± 0.00 ^a^
Monocytes (%)	0.00 ± 0.00 ^a^	0.00 ± 0.00 ^a^	0.00 ± 0.00 ^a^

Values are presented as mean ± standard error of the mean. Means in the same row as the control group with different superscript letters are significantly different. *p* < 0.05, Tukey HSD.

**Table 6:** Relative organ weight of the rats treated with BSO for 28 days.

Organ	Control	H-BSO	L-BSO
Male			
Heart	0.24 ± 0.01	0.25 ± 0.01	0.26 ± 0.01
Liver	3.05 ± 0.09	3.49 ± 0.22	3.20 ± 0.10
Kidney	0.63 ± 0.01	0.67 ± 0.02	0.65 ± 0.02
Lung	0.33 ± 0.01	0.34 ± 0.01	0.36 ± 0.01
Spleen	0.18 ± 0.00	0.19 ± 0.00	0.19 ± 0.00
Testis	0.91 ± 0.03	0.88 ± 0.04	0.90 ± 0.02
Female			
Heart	0.27 ± 0.01	0.26 ± 0.00	0.28 ± 0.01
Liver	3.21 ± 0.06	3.09 ± 0.04	3.17 ± 0.17
Kidney	0.66 ± 0.02	0.63 ± 0.01	0.69 ± 0.01
Lung	0.40 ± 0.01	0.40 ± 0.02	0.44 ± 0.03
Spleen	0.21 ± 0.01	0.27 ± 0.07	0.19 ± 0.01
Ovary and uterine tube	0.30 ± 0.03	0.33 ± 0.03	0.30 ± 0.02

Values are expressed as mean ± standard error of the mean (*n* = 6).

## Data Availability

Data are contained within the article.
